# De novo acute megakaryoblastic leukemia with p210 BCR/ABL and t(1;16) translocation but not t(9;22) Ph chromosome

**DOI:** 10.1186/1756-8722-4-45

**Published:** 2011-11-10

**Authors:** Xiao Min, Zhang Na, Liu Yanan, Li Chunrui

**Affiliations:** 1 Department of Hematology, Tongji Hospital of Tongji Medical College, Huazhong University of Science and Technology, 1095 Jie-Fang Avenue, Wuhan, Hubei, 430030, PR China

**Keywords:** Imatinib, Acute megakaryocytic leukemia, p210 BCR/ABL

## Abstract

Acute megakaryoblastic leukemia (AMKL) is a type of acute myeloid leukemia (AML), in which majority of the blasts are megakaryoblastic. De novo AMKL in adulthood is rare, and carries very poor prognosis. We here report a 45-year-old woman with de novo AMKL with BCR/ABL rearrangement and der(16)t(1;16)(q21;q23) translocation but negative for t(9;22) Ph chromosome. Upon induction chemotherapy consisting of homoharringtonine, cytarabine and daunorubicin, the patient achieved partial hematological remission. The patient was then switched to imatinib plus one cycle of CAG regimen (low-dose cytarabine and aclarubicin in combination with granulocyte colony-stimulating factor), and achieved complete remission (CR). The disease recurred after 40 days and the patient eventually died of infection. To the best of our knowledge, this is the first report of de novo AMKL with p210 BCR/ABL and der(16)t(1;16)(q21;q23) translocation but not t(9;22) Ph chromosome.

## Background

Acute megakaryoblastic leukemia (AMKL), also known as M7 under the French-American-British (FAB) classification, represents < 5% of acute myeloid leukemia (AML) [1-3]. In adults, AMKL constitutes only 0.5% - 1% of de novo AML cases [4]. Ph chromosome is a rare cytogenetic abnormality (≈ 1%) in AML [5,6]. The incidence of t(9;22) in AMKL varies considerably in the literature: from < 20% to > 60%, possibly due to inconsistency in the inclusion/exclusion of blastic phase of chronic myeloid leukemia (CML)[7-9].

Here, we report a case of 45-year-old woman with de novo AMKL. Multiple reverse transcription-polymerase chain reaction (RT-PCR) and Fluorescence in situ hybridization (FISH) data indicating a BCR/ABL rearrangement, cytogenetics for der(16)t(1;16)(q21;q23) but not t(9;22) Ph chromosome. Upon induction chemotherapy consisting of homoharringtonine, cytarabine and daunorubicin, the patient achieved partial hematological remission. The patient then received imatinib plus one cycle of CAG regimen (low-dose cytarabine and aclarubicin in combination with granulocyte colony-stimulating factor) [10], and achieved complete remission (CR). The disease recurred after 40 days and the patient eventually died of infection. The case diagnosis and management process, including the therapies, are summarized in Table 1.

**Table 1:** The clinical course of the patient

Time	Management process
**d1** **(3/20)**	**Diagnosis established;** **Induction chemotherapy consisting of homoharringtonine, cytarabine and daunorubicin began**.

**d30**	Bone marrow smear showed 20.4% megakaryoblasts and 24.8% promegakaryocytes (chemotherapy failure); Second induction chemotherapy.

**d60**	**Bone marrow smear showed 6% megakaryoblasts and 11% promegakaryocytes (partial remission);** **Imatinib treatment started (600 mg/d) and CAG regimen**

**d67**	WBC: 3.5 × 10^9^/L.

**d69**	**Imatinib reduced to 400 mg/d**.

**d75**	Imatinib discontinued (WBC: 0.9 × 10^9^/L).

**d102**	**Imatinib restarted (200 mg/d);** **WBC: 4.3 × 10^9^/L (d106)**

**d107**	Imatinib increased to 400 mg/d d110: complete hematological remission WBC:3.2 × 10^9^/L (d110); 3.5 × 10^9^/L (d120); 4.2 × 10^9^/L (d130)

**d132**	**Imatinib discontinued (due to financial reasons)**

**d150**	Relapse.

**d177**	**Patient deceased**.

Abbreviations: d, day; CAG regimen: cytarabine 30 mg/day for 14 days, aclarubicin 10 mg/day on days 1 - 8, and granulocyte colony-stimulating factor 300 μg/day on days 1 - 14; WBC, white blood cell count.

## Case Presentation

A 45-year-old woman was hospitalized on May 16th, 2008 with two weeks of fatigue, dizziness and low fever. The body temperature was 37.9°C. On auscultation, a II/VI systolic murmur was noticed over the apical region. The liver was palpable at 2 cm below the ribcage. The spleen was palpable at 2 cm below the left costal margin. Abdominal ultrasound confirmed slight hepatosplenomegaly. The patient had no history of toxic substance exposure. Family history was non-remarkable.

**Figure 1:**
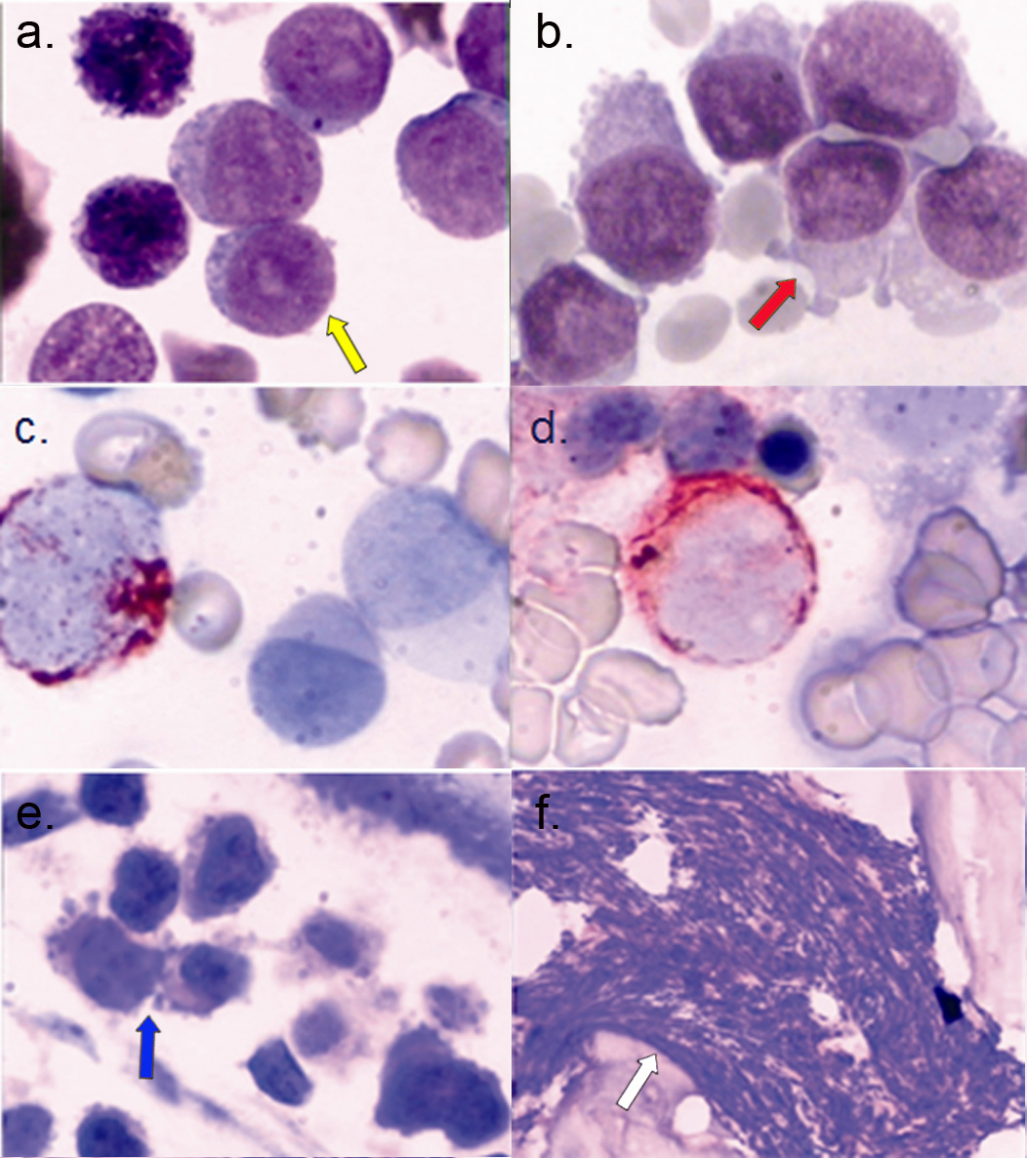
**Diagnosis of acute megakaryoblastic leukemia**. **a**. Bone marrow smear revealed many medium to large-size megakaryoblasts. The yellow arrow indicates megakaryoblasts (1000 ×). **b**. Bone marrow smear revealed many large-size promegakaryocytes. The cytoplasm of promegakaryocytes shows distinct pseudopod formation (1000 ×). The red arrow indicates the promegakaryocytes. **c, d**. Immunohistochemistry staining of CD41 (c) and CD42b (d) of bone marrow smears (1000 ×). The positivity was shown by the red precipitates in plasma or membrane. **e**. Bone marrow biopsy showing diffuse bone marrow infiltration of medium-size megakaryoblasts (1000 ×). The blue arrow indicates the megakaryoblasts. **f**. Bone marrow biopsy showing myelofibrosis (100 ×). The white arrow indicates myelofibrosis.

Blood tests revealed a hemoglobin concentration of 63 g/L, a hematocrit of 23%, a platelet count of 138 × 10^9^/L. White blood cell count was 24 × 10^9^/L, with 54% megakaryoblasts, 17% promegakaryocytes, 10% myelocytes, 8% band forms, 7% neutrophils, 3% lymphocytes, and 1% monocytes. Plasma D-dimer and lactate dehydrogenase (LDH) were normal. A bone marrow smear showed 67.2% megakaryoblasts and 20.4% promegakaryocytes. The megakaryoblasts were medium to large-size with round, slightly irregular nuclei and one to three nucleoli (Figure 1a). The cytoplasm of promegakaryocytes was basophilic and might show distinct pseudopod formation (Figure 1b). Immunohistochemistry staining (Leukocyte Phenotyping Kit, Sun BioTech, China) of these cells revealed a total of 55% positivity for CD41 (Figure 1c) and 60% positivity for CD42b (Figure 1d), while CD13, CD14, CD68, MPO, HLA-DR, CD10, CD19, CD3, CD5, and CD7 were all negative A bone marrow biopsy indicated acute leukemia with myelofibrosis (Figure 1e &1f).

**Figure 2:**
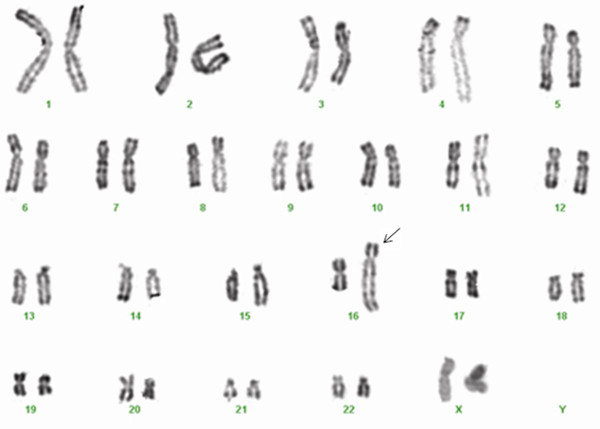
**Representative karyogram of bone marrow cell showing the 46,XX,der(16)t(1;16)(q21;q23)**.

**Figure 3:**
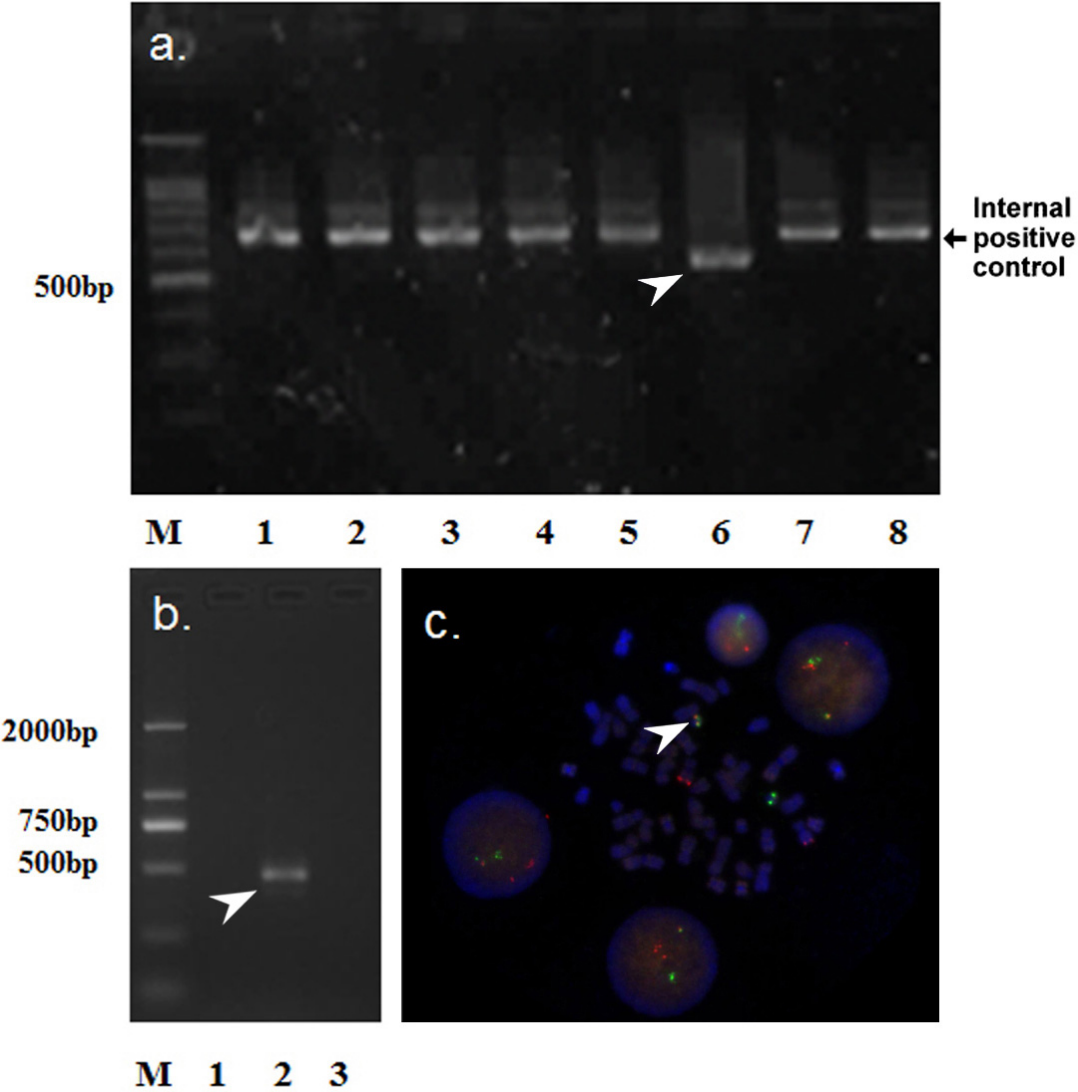
**Detection of fusion genes**. **a**. Multiplex reverse transcription-PCR analysis showed that the cells of this patient were positive for a translocation in multiplex reaction 6 (The arrow). Internal positive control: E2A (690 bp). The primer sets used in multiplex reverse transcription-PCR were chosen as described previously [11] to detect the following fusion genes: 1. CBFβ/MYH11/MLL/AFX1/AF6/ELL/E2A; 2. MLL/AF1P/AF17/AF10/E2A; 3. PBX1/SIL/TAL1/HLF/TEL/AML1A/E2A; 4. AML1A/AMLMDSEVI/HOX11/ETO/TLS/ERG/E2A; 5. MLL/AF4/AF9/AF1Q/ENL/E2A; **6. BCR/ABL/TEL/E2A; **7. DEK/CAN/SET/AMLMDSEVI /E2A; 8. PLZF/PML3/RARα/NPM/NPMALK /MLF1/E2A; **b**. The split-out PCR analysis revealed a BCR/ABL b2a2 fusion gene expression using primer sets for 1. BCR/ABL e1a2 (320 bp); **2. BCR/ABL b2a2 (472 bp) **and BCR/ABL b3a2 (397 bp); 3. TEL/ABL (366 bp); **c**. Fluorescence in situ hybridization (FISH) using a Vysis LSI BCR/ABL Dual Color, Dual Fusion Translocation Probe (Vysis, Downers Grove, IL, USA) on an interphase cell. Orange signal is ABL gene on chromosome 9 and green signal is BCR gene on chromosome 22. The ABL orange signals occurred on both chromosomes 9 and on der(9)ins(22;9) and one BCR green signal on chromosomes 22 and one yellow fusion signal on der(22)ins(22;9). The arrow indicates the fusion signal.

Cytogenetic analysis of trypsin R-banded chromosome preparations revealed 46, XX, der(16)t(1;16)(q21;q23)[8]/46,XX [12] (Figure 2). To identify fusion genes, multiplex reverse transcription-polymerase chain reaction (RT-PCR) was performed with 1~8 parallel nested (2-round) multiplex reactions in a thermocycler (Perkin-Elmer) to achieve maximal sensitivity, as described in a previous study [11]. The E2A mRNA was used as the internal positive control. The groups containing possible fusion genes were further characterized using split-out PCR to identify the fusion pattern as described previously [11]. The results suggested the presence of fusion among the following genes: BCR, ABL and TEL (Figure 3a). A split-out PCR analysis was performed using the individual primer sets BCR/ABL e1a2, BCR/ABL b2a2 or b3a2, TEL/ABL. The results revealed fused BCR/ABL b2a2 mRNA expression (Figure 3b). FISH analysis on interphase cells revealed an atypical signal pattern consisting of one green signal, two orange signals, and one orange/green (yellow) fusion signal in approximately 30% of the cells (Figure 3c).

On the basis of the above reported clinical and biological features, a diagnosis of de novo acute AMKL. The patient received induction had regimen consisting of: homoharringtonine (2 mg/m^2^/day on day 1 - 7), cytarabine (100 mg/m^2^/day on day 1 - 7) and daunorubicin (45 mg/m^2^/day on day 1 - 3). A bone marrow smear at one month later showed no improvement. A partial remission was achieved after the induction treatment was repeated. The patient then received imatinib (600 mg/d, p.o.) and one cycle of CAG regimen (cytarabine 30 mg/day for 14 days, aclarubicin 10 mg/day on days 1 - 8, and granulocyte colony-stimulating factor 300 μg/day on days 1 - 14). Imatinib was discontinued after 2 weeks due to severe bone marrow suppression. Plasma LDH and liver enzymes remained within the normal range during the treatment. A complete hematological response was achieved upon evaluation at 50 days after initiating imatinib treatment, and the patient was discharged. She was hospitalized for high fever and dyspnea after 40 days. Hemoglobin was 90 g/L. White blood cell count was 19 × 10^9^/L, with 21% blast cells. Relapse was established with bone marrow smear. The patient was treated with cytarabine (2 g/m^2^/day on days 1 - 3) and daunorubicin (45 mg/m^2^/day on days 1 - 3), with no apparent improvement. She died of fungal infection after 27 days.

## Conclusions

Although the first AMKL was described as early as 1931, reports have been sporadic because of both the rarity of the disease and the lack of well-established diagnostic criteria. In fact, precise diagnostic criteria were added to the French-American-British classification only in 1985 (FAB M7) [1]. The bone marrow aspirate shows a leukemic cell infiltrate that comprises 30% or more of all cells. These cells are identified as being of megakaryocyte lineage by platelet peroxidase reaction on electron microscopy or by tests with monoclonal or polyclonal platelet-specific antibodies (CD41a, CD42 or CD61). Myelofibrosis or increased bone marrow reticulin are a prominent aspect in most AMKL patients. In some cases, megakaryoblastic crisis could be the first presentation of CML, and not distinguishable from de novo AMKL [12-16]. This case represents de novo AMKL in our opinion, because the patient had no basophilia and eosinophilia upon presentation, which are often seen in blast crisis of CML [17]. Basophilia, a frequent feature of blast crisis, is uncommon in acute leukemias [18]. Furthermore, our patient had only mild splenomegaly. Moderate or severe splenomegaly is common in blast crisis of CML [13,16].

AMKL is associated with no specific cytogenetic abnormality, and the majority of cases present with a complex karyotype. In a recent study by Le Groupe Francais de Cytogenetique Hematologique (GFCH), complex karyotypes of unbalanced changes, such as -5/del5q or -7/del(7q), 3q21q26, dic(1;15)(p11;p11), inv(4)(p15q11), t(14;21)(q24;q22), der(7)t(7;17)(q11;q11) and t(6;13)(p22;q14), were common in adult de novo AMKL [8]. In addition, the t(X;16) translocation has also been reported in 2 adult de novo AMKL cases [8]. One translocation was 46,XY,t(16;21)(p11;q22), another was t(16;16)(p13;q22). The present case serves to identify a novel translocation der(16)t(1;16)(q21;q23), providing further insight into the heterogeneity of genomic rearrangement in this subset of AML.

Data concerning the incidence of the Ph chromosome or BCR/ABL rearrangement in de novo AMKL are scarce. The Ph chromosome is one of the most common chromosomal abnormalities associated with adult AMKL according the report of the GFCH [8]. For example, Ph chromosome was found in four out of a total of 23 AMKL cases (17%) [8]. In fact, only two cases were de novo AMKL (9%). In an early study of 14 AMKL patients with cytogenetic data, Ph chromosome was found in two cases of megakaryoblastic transformation of chronic myelogenous leukemia, but not in de novo AMKL [7]. Ohyashiki et al. reported three cases with AMKL, but none had Ph chromosome [9].

Reports of Ph chromosome or BCR/ABL rearrangement are summarized in Table 2. Cases were heterogeneous and the survival was from 1.9 to 96 months. The case reported by Kaloutsi et al. [20] was a 24-year-old male with de novo AMKL. Interestingly, cytogenetics revealed a t(10;22), which by FISH, was found to be a variant Philadelphia translocation involving chromosome 10q. The FISH result in our case revealed an atypical signal pattern: one yellow fusion signal with one green and two orange signals (Figure 3c). This result confirmed that the detected variant translocation involved fragments of two chromosomes: 9 and 22. The ABL orange signals occurred on both chromosomes 9 and on der(9)ins(22;9) and one BCR green signal on chromosomes 22 and one yellow fusion signal on der(22)ins(22;9).

**Table 2:** Literature review of Ph chromosome or BCR/ABL rearrangement in de novo acute megakaryoblastic leukemia

Case No.	Sex/Age (years)	Cytogenetic changes	BCR/ABL fusion transcripts	Survival (month)	Reference
**1**	**F/58**	**47,XX,+8,t(9;22)(q34;q11)**	**P190 BCR/ABL**	**6**	**Balatzenko, et al**[19]

**2**	F/72	46,XX,t(9;22)(q34;q11)[12]/(4n) 92,XXXX,t(9;22)x2 [10]/(8n)184,XXXXXXXX,t(9;22)x4 [4]	Not provided	27	Dastugue, et al [8]

**3**	**F/51**	**46,XX,t(9;22)(q34;q11)[25]**	**Not provided**	**27**	**Dastugue, et al **[8]

**4**	F/44	46,XX,inv(3)(q21q26)[4]/46,idem,t(9;22)(q34;q11)[15]	Not provided	1.9	Dastugue, et al [8]

**5**	**M/24**	**46,XY,t(10;22)(q26;q11)** **FISH: t(9;22);10q**	**Not provided**	**Not provided**	**Kaloutsi, et al **[20]

**6**	M/53	46,XX,t(9;22)(q34;q11),del(18)(p10)[15]	BCR/ABL e6a2	96	Corm, et al [21]

**7**	**M/39**	**t(9;22)(p24.1;q12.2)** **t(8;17)(q23;q24.2).**	**Not provided**	**Not provided**	**Ahmad, et al **[22]

To the best of our knowledge, this is the first report of de novo AMKL with rare variant of Philadelphia rearrangement and a novel translocation der(16)t(1;16)(q21;q23). Our case and the case reported by Kaloutsi et al. [20] suggested that the FISH should be considered for detection of variant Philadelphia rearrangement in de novo AMKL patients.

## Consent

Written informed consent was obtained from the husband of the patient for publication of this case report and any accompanying images. A copy of the written consent is available for review by the Editor-in-Chief of this journal.

## Competing interests

The authors declare that they have no competing interests.

## Authors' contributions

XM was responsible for data acquisition and analyses, as well as data interpretation. ZN participated in manuscript preparation and contributed significantly to the concept development. LYN participated in patient management, and also contributed to data interpretation. LCR was responsible of patient management and conceived the study. All authors read and approved the final manuscript.
